# Mediastinal large B cell lymphoma and surrounding gray areas: a report of the lymphoma workshop of the 20th meeting of the European Association for Haematopathology

**DOI:** 10.1007/s00428-023-03550-5

**Published:** 2023-08-02

**Authors:** Sarah E. Gibson, Stefan Dojcinov, Snjezana Dotlic, Sylvia Hartmann, Eric D. Hsi, Monika Klimkowska, Federica Melle, Stefano A. Pileri, Colleen A. Ramsower, Karen Rech, Lisa M. Rimsza, Socorro Maria Rodriguez-Pinilla, Thomas A. Tousseyn, Daphne de Jong, Elena Sabattini

**Affiliations:** 1 Division of Hematopathology, Department of Laboratory Medicine and Pathology, Mayo Clinic, Phoenix, AZ USA; 2 Department of Pathology, Morriston Hospital, Swansea Bay University Health Board, Swansea, UK; 3 Department of Pathology and Cytology, University Hospital Centre Zagreb, Zagreb, Croatia; 4 Dr. Senckenberg Institute of Pathology, Goethe University Frankfurt Am Main, Frankfurt Am Main, Germany; 5 Department of Pathology, Wake Forest University School of Medicine, Winston-Salem, NC USA; 6 Department of Clinical Pathology and Cancer Diagnostics, Karolinska University Hospital, Stockholm, Sweden; 7 Division of Haematopathology, IEO, European Institute of Oncology IRCCS, Milan, Italy; 8 Department of Research, Mayo Clinic, Scottsdale, AZ USA; 9 Department of Laboratory Medicine and Pathology, Mayo Clinic, Rochester, MN USA; 10 Pathology Department, Hospital Universitario Fundación Jiménez Díaz, Madrid, Spain; 11 Department of Imaging and Pathology, Translational Cell and Tissue Research Lab, KU Leuven, Louvain, Belgium; 12 Department of Pathology, Amsterdam UMC, Location VUMC, De Boelelaan 1117, 1081HV Amsterdam, The Netherlands; 13 Haematopathology Unit, IRCCS Azienda Ospedaliero-Universitaria Di Bologna, Bologna, Italy

**Keywords:** Primary mediastinal large B cell lymphoma, Classic Hodgkin lymphoma, Gray zone lymphoma, Mediastinal gray zone lymphoma, Composite lymphoma, B cell lymphoma, Unclassifiable, with features intermediate between diffuse large B cell lymphoma and classic Hodgkin lymphoma

## Abstract

Session 3 of the 2021 European Association for Haematopathology/Society for Hematopathology Workshop focused on mediastinal large B cell lymphomas and surrounding gray areas. One half of the session was dedicated to primary mediastinal large B cell lymphoma (PMBL) and included cases with classic clinicopathologic features, as well as cases with either morphologic or immunophenotypic variation, and PMBL-like cases with primary extramediastinal disease. The role of additional immunophenotyping and/or molecular testing to aid in the diagnosis of PMBL was discussed. The second half of the session focused on mediastinal and non-mediastinal gray zone lymphomas (GZL) with features intermediate between diffuse large B cell lymphoma (DLBCL) and classic Hodgkin lymphoma (CHL). Several cases illustrating the current challenges in separating this entity from PMBL/DLBCL and CHL were presented. There was discussion regarding the clinical and genetic differences between mediastinal and non-mediastinal GZLs. Rare cases of PMBL and GZL associated with EBV or follicular lymphoma were reviewed. Finally, several cases included in the session highlighted composite or sequential CHL and PMBL/DLBCL and/or GZL, highlighting challenges in separating such cases from GZL.

**Supplementary Information:**

The online version contains supplementary material available at 10.1007/s00428-023-03550-5.

## Introduction

The distinct thymic niche of the mediastinum gives rise to a well-described spectrum of B cell lymphomas, with primary mediastinal (thymic) large B cell lymphoma (PMBL) resting at one end of the spectrum and classic Hodgkin lymphoma (CHL), particularly of the nodular sclerosis (NS) subtype, resting at the other [1, 2]. Mediastinal gray zone lymphomas (MGZL), previously known as B cell lymphoma, unclassifiable, with features intermediate between diffuse large B cell lymphoma (DLBCL) and CHL (BCLU) in the revised 4th edition of the World Health Organization (WHO-HAEM4R) classification [2], span the biologic spectrum between these two well-defined entities, with some cases more closely resembling PMBL and others more closely resembling CHL [1, 2]. Although these diagnostic entities are well-established, in routine clinical practice, there remain practical challenges to their diagnosis, particularly with the diagnosis of gray zone lymphoma (GZL) [3]. The routine immunophenotyping and/or molecular testing required for PMBL and GZL diagnoses also varies amongst institutions [4–10]. In addition, there are increasing reports of mediastinal-type large B cell lymphomas (LBCL) and GZLs arising at non-mediastinal sites, which raises further questions on how these lymphomas should be diagnosed and classified [2, 11–15].

In recognition of these diagnostic challenges and emerging questions, one lymphoma workshop session of the 20th meeting of the European Association for Haematopathology (EAHP-WS) held in April 2021 was dedicated to mediastinal large B cell lymphoma and surrounding gray areas. One part of the session was comprised of 18 cases exemplifying PMBL and its diagnostic borders, including PMBLs that had classic clinicopathologic features, as well as unusual cases showing either morphologic or immunophenotypic variability, association with Epstein-Barr virus (EBV), or extramediastinal disease. The second part of the session included 36 cases that illustrated various aspects of GZL, highlighting the challenges in separating this entity from PMBL/DLBCL, CHL, and EBV-positive DLBCL. Nine cases included in this session also represented composite synchronous or sequential lymphomas, illustrating the difficulty in separating true composite CHL and LBCL cases from GZL.

From the case series submitted to this session of the EAHP-WS, five major topics will be considered in this report relating to (1) PMBL, (2) EBV-negative GZL, (3) EBV significance in mediastinal B cell lymphomas with PMBL or MGZL features, (4) PMBL or GZL as transforming or associated events in follicular lymphoma (FL), and (5) composite or sequential CHL and PMBL/DLBCL and/or GZL. It should be noted that after the EAHP-WS was held in 2021, updates were made to the classification of GZLs with the publication in 2022 of the International Consensus Classification (ICC) and 5th edition of the WHO Classification of Lymphoid Neoplasms (WHO-HAEM5) [16, 17]. Both classifications now prefer the term MGZL over what was previously designated BCLU in the WHO-HAEM4R classification [2], restricting the GZL diagnosis to only those lymphomas arising in the mediastinum. It is suggested that lymphomas with GZL-like features outside of the mediastinum should instead be designated as DLBCL, NOS, or EBV-positive DLBCL [16, 17]. This concept is in keeping with the major points of discussion at the EAHP-WS and is further retained in this report.

## PMBL and its diagnostic borders

### Background

PMBL represents a distinct subtype of LBCL initially recognized in the 1980s and formally included in the Revised European-American Classification of Lymphoid Neoplasms in 1994 and subsequent WHO classifications [2, 18, 19]. Although PMBL has morphologic features in keeping with a DLBCL, it has clinical, gene expression (GE), and genetic overlap with CHL [2, 20, 21]. Clinically, PMBL has a predominant presentation in young females, localization to the thymic area in the anterosuperior mediastinum, and a lack of bone marrow or other disseminated disease at presentation [2]. Microscopically, PMBL is frequently associated with compartmentalizing fibrosis and the neoplastic B cells are medium to large in size with abundant pale cytoplasm and have round or ovoid nuclei, or in some cases, multilobated or even Hodgkin/Reed-Sternberg (HRS)-like nuclei [2]. PMBL shows expression of pan-B cell antigens, commonly lacks immunoglobulin expression, and stains for CD30 in > 80% of cases, although the degree and intensity of staining are variable [2, 22]. CD23, MAL, CD200, PDL1, and PDL2 are positive in ≥ 70% of cases [2, 5, 6, 9, 22]. CD15 is usually negative, although a paranuclear dot-like pattern of expression can still be consistent with an otherwise classic PMBL [23]. PMBL has a gene expression (GE) signature that is unique among LBCLs, but does overlap with that of CHL, including activation of NF-κB and JAK/STAT signaling pathways and reduced B cell receptor signaling [2, 20–22, 24]. Although GE analysis of PMBL has previously relied on frozen tissue, recently developed assays using routine formalin-fixed tissue specimens are available at limited institutions [4, 8]. Recurrent genomic alterations found in PMBL support NF-κB and JAK/STAT activation, as well as an immune privileged phenotype [2, 20, 21]. Common alterations leading to constitutive NF-κB activation include gains of chromosome 2p16.1/*REL* (approximately 50% of cases) and inactivating mutations of *TNFAIP3* (up to 60% of cases) and *NFKBIE* (up to 30% of cases) [2, 20, 21, 25]. Constitutive activation of the JAK/STAT pathway, which is also a feature of CHL, is related to inactivating mutations of *SOCS1* (up to 60% of cases) and *PTPN1* (approximately 25% of cases), gain of function mutations in *STAT6* (up to 70% of cases), and recurrent copy number gains/amplification of 9p24.1/*JAK2* (> 70% of cases) [2, 20, 21]. Chromosome 9p24.1 also contains the *PDL1/PDL2* locus, which along with rearrangements and/or mutations of *CIITA* (> 50% of cases) and alterations of *B2M* (up to 40% of cases) and *CD58* (up to 20% of cases), provide mechanisms for immune escape [2, 20, 21].

### Discussion of cases with classic features of PMBL submitted to the workshop

**Fig. 1:**
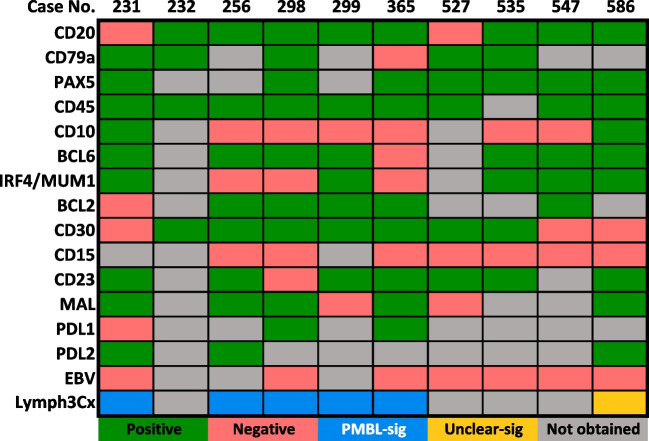
Immunophenotype and Lymph3Cx analysis of 10 Workshop cases with features of classic PMBL. The figure illustrates the immunophenotype of each classic PMBL case, as well as the results of Lymph3Cx analysis. Abbreviations: No., number; PMBL-sig, primary mediastinal large B-cell lymphoma gene expression signature; Unclear-sig, unclear diffuse large B-cell lymphoma versus primary mediastinal large B-cell lymphoma gene expression signature

Ten cases submitted to the workshop met criteria for classic PMBL (cases 231, 232, 256, 298, 299, 365, 527, 535, 547, and 586). These patients included six females and four males with a median age of 26 years (median 12–48 years), and all patients presented with an anterior mediastinal mass. Four of these cases also had non-regional, extramediastinal disease at presentation, including involvement of axillary lymph nodes (cases 256 and 535), inguinal lymph nodes (case 298), and pancreas (case 231). Although these cases all expressed CD45 and at least one pan-B cell antigen (unfortunately, a complete set of B-markers was not available in all cases), 20% lacked CD20 expression, a rarely described phenomenon in PMBL (Fig. 1) [26, 27]. The lack of CD20 expression in these rare PMBLs may create diagnostic confusion with CHL or GZL, particularly if the neoplastic cells also exhibit strong CD30 expression; therefore, it is advisable in such cases to assess additional pan-B cell antigens such as PAX5, CD79a, OCT2, BOB1, or CD19. In accordance with what has been previously described in the literature, most of the classic PMBL cases in this session showed expression of CD30 (70%), CD23 (88%), MAL (71%), PDL1 (67%), and PDL2 (100%), although the number and intensity of positive cells varied (Supplementary Fig. 1). CD200 was not assessed in this group of cases. No rearrangements of *MYC*, *BCL2*, or *BCL6* were identified in the five cases tested. A rearrangement of *CIITA* and copy number gain of the *PDL1/PDL2* locus were identified in the one case that was tested (case 231). The Lymph3Cx assay, which discriminates between DLBCL and PMBL and assigns cell-of-origin group on the basis of GE, was performed on six cases in this group as previously described (Fig. 1) [8]. Five of the six cases showed a PMBL signature, while one case (586, from D. Olson, Minnesota Childrens Hospital, USA), arising in a 15-year-old female with an isolated mediastinal mass, showed an unclear GE signature between PMBL and DLBCL. However, the Lymph3Cx probability score for this case (0.86) was very close to the cutoff to call PMBL (≥ 0.90), and otherwise, this case was morphologically compatible with PMBL, expressed CD23, MAL, and PDL2, but did lack CD30 expression.

### Cases illustrating challenges to defining the diagnostic border between PMBL and DLBCL

**Fig. 2:**
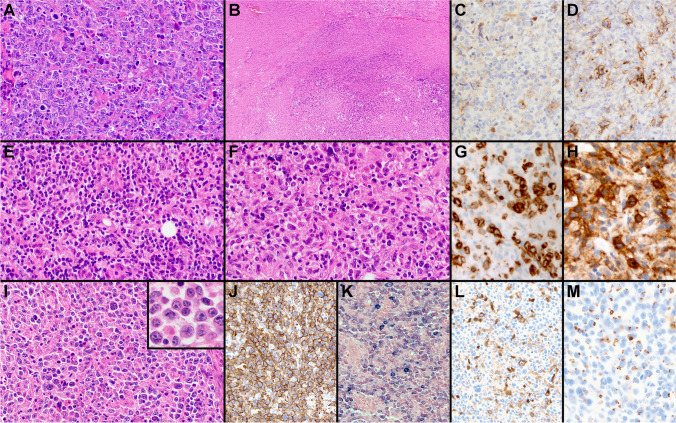
Mediastinal B-cell lymphomas challenging the diagnostic border between PMBL and DLBCL (cases 489, 162, and 264). Case 489 showed centroblastic cytology (**A**), extensive necrosis (**B**), and only focal CD30 (**C**) and CD23 (**D**) expression. Case 162 demonstrated features reminiscent of a T cell/histiocyte-rich LBCL (**E**–**F**), with expression of CD79a (**G**) and CD30 (**H**). Case 264 showed immunoblastic and plasmacytoid cytology (**I**). The neoplastic cells were positive for CD20 (**J**), with kappa light chain restriction (**K**), rare CD30 expression (**L**), and showed diffuse Golgi/dot-like staining for CD15 (**M**)

There were several cases submitted to the workshop that challenged the distinction between PMBL and DLBCL (cases 162, 193, 251, 264, and 489). They were all EBV negative. The first set of these cases had morphologic and/or immunophenotypic features that were not entirely typical for PMBL, but showed GE and/or other genetic features supporting their classification as PMBL. Case 489 (A. Nicolae, Hautepierre Hospital, France), presenting as an anterior mediastinal mass in an 18-year-old male, showed predominantly centroblastic cytology and extensive necrosis (Fig. 2A–D). Although the LBCL demonstrated only focal expression of CD23 and PDL1, and rare CD30 staining, RT-MLPA analysis [4] showed a PMBL GE signature and sequencing studies showed mutations in *SOCS1* and *STAT6*, supporting classification of this LBCL as PMBL. Case 162 (A. Davis, Hospital of the University of Pennsylvania, USA), presenting as a mediastinal mass in a 65-year-old male, was quite polymorphous, consisting of scattered large B cells admixed with numerous histiocytes and small lymphocytes, reminiscent of a T cell/histiocyte-rich LBCL, although focal compartmentalizing fibrosis was present (Fig. 2E–H). The large B cells expressed CD30 and PDL1, but lacked CD23 and MAL staining. Although the morphologic and immunophenotypic features in this case were not entirely in keeping with PMBL, Lymph3Cx analysis confirmed a PMBL GE signature and sequencing studies showed mutations in *GNA13*, *SOCS1*, *TNFAIP3*, and *XPO1*, compatible with classification as PMBL. The last case in this group, case 264 (S. Weindorf, Stanford University, USA), presented in a 62-year-old female with an anterior mediastinal mass, as well as lymphadenopathy involving cervical, abdominal, and retroperitoneal regions. The LBCL had a striking immunoblastic and plasmacytoid appearance (Fig. 2I–M). The neoplastic cells strongly expressed CD20, were dim kappa light chain restricted with in situ hybridization stains, showed only rare CD30 positivity, but had diffuse golgi/dot-like staining for CD15. The large B cells also partially expressed CD45, CD23, and CD200, and were negative for MAL, CD138, MNDA, IgM, ALK1, HHV8, and EBER. Fluorescence in situ hybridization (FISH) studies showed an isolated *MYC* rearrangement, and Lymph3Cx analysis showed a PMBL GE signature. Although the diagnosis of this case remains debatable given its unusual morphology and immunophenotype, the GE profile and mediastinal presentation do raise the possibility that this lymphoma falls within the PMBL spectrum. Other ancillary studies such as FISH or sequencing analysis to evaluate for additional PMBL-associated genetic alterations might be helpful to definitively classify unusual mediastinal B cell lymphomas such as this.

**Fig. 3:**
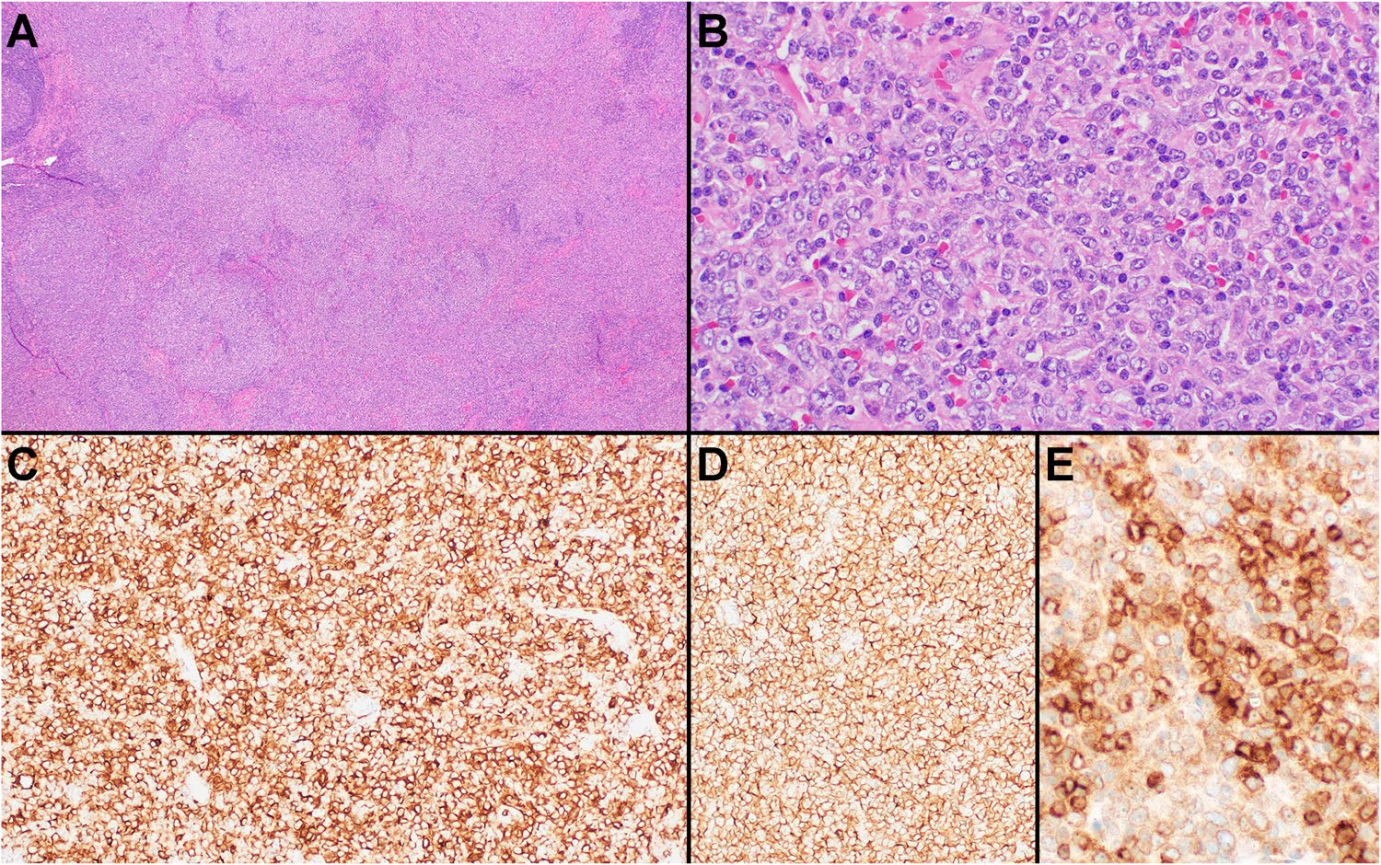
Extramediastinal transformed FL with a PMBL gene expression signature (case 193). Grade 3A of 3 FL (**A**) with a component of LBCL (**B**). The large B cells expressed CD10 (**C**), CD23 (**D**), and MAL (**E**)

The second set of cases included in this group included two extramediastinal LBCLs with PMBL-like features. In recent years, DLBCLs with a PMBL-like GE or genetic features, such as chromosome 9p24.1 amplification, have been increasingly recognized at non-mediastinal locations [11–15]. Case 193 (L. Rimsza, Mayo Clinic Scottsdale, USA) is an example of one of these rare lymphomas, but expands the description of such cases to the setting of transformed FL. This 72-year-old female had a history of previously treated FL and developed new right inguinal lymphadenopathy, which showed a LBCL associated with grade 3A of 3 FL (Fig. 3). The LBCL had mostly centroblastic cytology, only showed rare CD30 positive cells, but was strongly positive for CD10, CD23, and MAL. Although this LBCL was morphologically and immunophenotypically compatible with a DLBCL of germinal center B cell (GCB) type, Lymph3Cx analysis surprisingly showed a PMBL GE signature. In contrast, case 251 (T. Tousseyn, UZ Leuven, Belgium) had morphologic and immunophenotypic features suggesting a non-mediastinal PMBL, which was not born out on the molecular level. This LBCL presented in a 25-year-old female with abdominal and retroperitoneal lymphadenopathy and no mediastinal disease, and showed morphologic and immunophenotypic features reminiscent of PMBL including compartmentalizing fibrosis and strong expression of CD30, CD23, PDL1, MAL, and CD200. Although these morphologic and immunophenotypic findings strongly suggested a non-mediastinal PMBL, Lymph3Cx analysis indicated an unclear GE signature between PMBL and DLBCL with a probability score (0.10) that was very close to the cutoff for DLBCL (< 0.10). Cytogenetic studies showed a mild amplification of *REL*, but no evidence of *PDL1* gain/amplification or rearrangements of *MYC*, *BCL2*, or *BCL6*, and sequencing studies demonstrated mutations in *EZH2* and *EP300*, a genetic profile more in keeping with the GCB or EZB subtype of DLBCL [28]. Previous studies of non-mediastinal DLBCLs with PMBL GE or genetic features indicate that such cases morphologically resemble PMBL and frequently express CD23, MAL, or PDL1 [12, 14]. However, none of these features seem highly sensitive or specific, and cases 193 and 251 illustrate that these lymphomas cannot always be predicted by morphology or immunophenotype alone. Whether more routine cytogenetic, sequencing, and GE studies are indicated to identify these rare non-mediastinal PMBLs is not entirely clear at this time.

## (Mediastinal) GZL and its diagnostic borders

### Background

GZL was first identified as a distinct entity in the 2008 WHO classification, and was defined as a BCLU in the WHO-HAEM4R classification [2, 29]. The disease was initially described as primarily arising in the mediastinum, which remains the most common site of onset in 70–80% of cases, although rare non-mediastinal presentations have been suggested to occur [2, 30–33]. MGZLs tend to occur in young patients (29.5 to 39 years), more commonly in males, with more frequent localized, yet often bulky, disease [2, 30–33]. The few non-mediastinal GZL cases described seem to differ in some clinicopathologic features. Patients with non-mediastinal disease tend to be older than 50 years of age, are more commonly female (M:F ratio of 3:6), and present at more advanced clinical stage, with less bulky disease and more common extranodal involvement [1]. Recent studies have shown differences in the GE profile and genomic alterations between MGZLs and those GZLs arising outside the thymic niche [34, 35]. For these reasons, both WHO-HAEM5 and ICC classifications narrow the definition of GZL to include only those cases arising in the mediastinum, replacing the category of BCLU with MGZL [16, 17]. It is proposed that lymphomas showing morphologic and immunophenotypic features consistent with GZL that occur outside of the mediastinum are better classified as DLBCL, NOS [16, 17].

GZLs include cases with predominant CHL-like, PMBL/DLBCL-like, or intermediate/transitional morphologic features that show immunophenotypic discrepancies beyond the tolerated degree of variability for each specific diagnostic category [1, 2, 30–33]. A more detailed morphologic subclassification was recently reported by Sarkozy et al.: LYSA group 0 (CHL morphology and full strong expression of all B cell markers), LYSA group 1 (CHL-like with possible transitional and PMBL-like areas, variable expression of B cell markers), LYSA group 2 (predominant PMBL-like morphology, but still too rich in inflammatory cells and HRS cells), and LYSA group 3 (substantial overlap with PMBL, with immunophenotypic anomalies such as diffuse, strong CD15 and/or CD30 expression) [1]. Despite these notable attempts, the diagnosis remains challenging in routine clinical practice, with significant implications for therapy choice, given that patients with GZL show inferior survival rates compared with DLBCL or CHL regardless of the type of administered therapy [36]. Two recent papers evaluated the issue of the reproducibility of the diagnosis of GZL using a central pathology consensus case review [1, 3]. Not unexpectedly, only 38% [3] and 55% [1] of cases were confirmed as GZL, the remaining being reclassified as NSCHL (syncytial or NS2 type), lymphocyte-predominant Hodgkin lymphoma, DLBCL, NOS, EBV-positive DLBCL, T cell/histiocyte-rich LBCL, or PMBL, which represent potential misdiagnoses that need to be excluded before making the diagnosis of GZL.

Regarding immunophenotype, most reports focus on the expression of CD30, CD15, strong PAX5, and CD20, the latter marker often being the pivotal parameter for this diagnosis. CD30 is commonly expressed diffusely and strongly, closer to the staining observed in CHL than in PMBL, while expression of CD15 is mostly felt to favor a GZL diagnosis if expressed diffusely and strongly [2, 30]. Only limited genetic data for GZL has been available until recently, when three major publications shed light on this topic both at the GE and genetic levels [34, 35, 37]. The GE of MGZL lies within the spectrum of signatures seen in CHL and PMBL, with the CHL-like/LYSA group 0–1 cases clustering closer to CHL samples and the LBCL-like/LYSA group 2–3 cases closer to the PMBL samples [34, 37]. Particularly, LYSA-defined group 3 cases show substantial GE overlap with the PMBL signature [34]. The Lymph3Cx assay mostly provides a PMBL signature in these cases [34]. Mutational profiling of MGZL shows a mutation pattern similar to that seen in CHL and PMBL, including recurrent mutations of *SOCS1*, *B2M*, *TNFAIP3*, *GNA13*, and *NFKBIA* [35]. Chromosomal abnormalities, including gains/amplification of 2p16.1/*REL*, alterations of 9p24.1/*JAK2*/*PDL1*/*PDL2*, and rearrangements of 16p13.13/*CIITA,* are also common in MGZL [38]. In contrast, the GE profile of a subset of non-mediastinal GZLs seems closer to that seen in DLBCL rather than PMBL [34]. Non-mediastinal GZLs are also enriched in mutations related to the apoptotic pathway and may harbor an increased frequency of *BCL6* rearrangement [35], findings that question whether these lymphomas should be considered within the spectrum of MGZL or rather classified separately as DLBCL with GZL-like features. These observations support the concept of mediastinal B cell lymphomas as a group of biologically related diseases, leading to recent classification updates.

### Discussion of cases submitted to the workshop as GZL

**Fig. 4:**
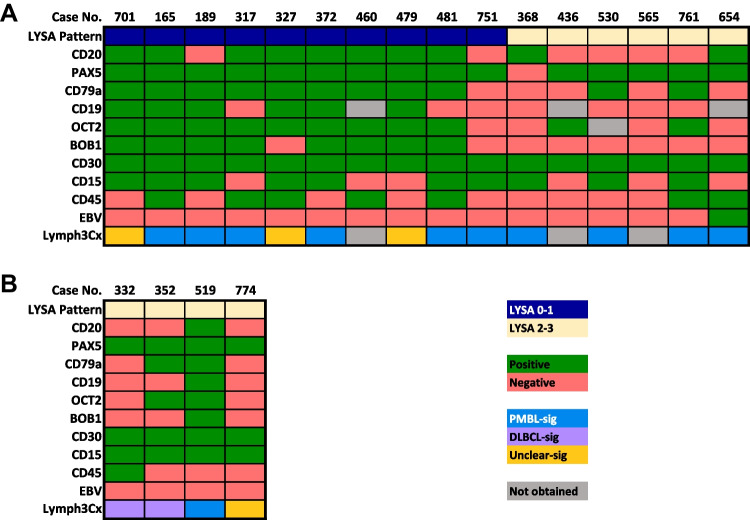
Immunophenotype and Lymph3Cx analysis of 20 Workshop cases submitted as GZL. Sixteen MGZLs (15 EBV negative and one (case 654) EBV positive) (**A**) and four EBV-negative non-mediastinal cases (**B**) are illustrated. The morphologic classification of these cases into CHL-like/LYSA groups 0–1 or LBCL-like/LYSA groups 2–3 is included. Abbreviations: DLBCL-sig, diffuse large B-cell lymphoma gene expression signature; No., number; PMBL-sig, primary mediastinal large B-cell lymphoma gene expression signature; Unclear-sig, unclear diffuse large B-cell lymphoma versus primary mediastinal large B-cell lymphoma gene expression signature

**Fig. 5:**
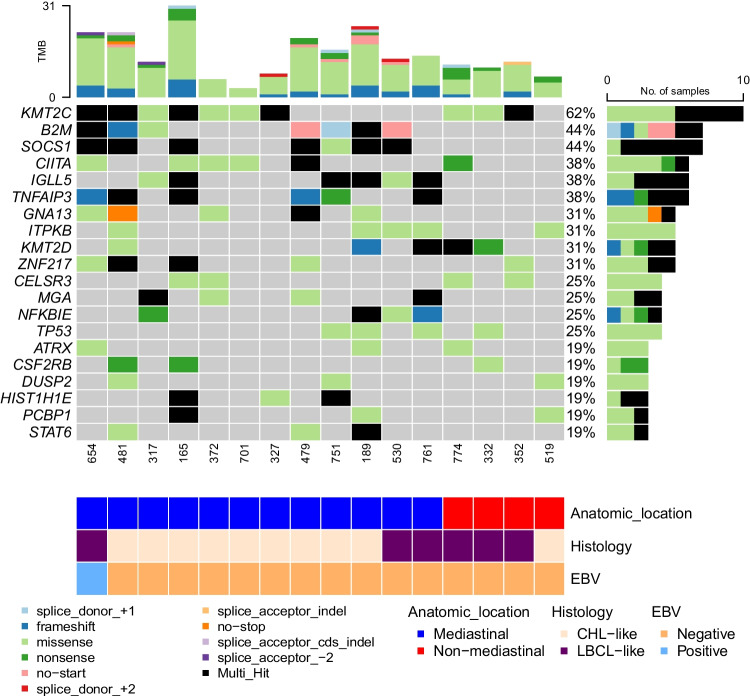
Next-generation sequencing analysis of 16 workshop cases submitted as GZL. Alterations found in 12 MGZLs [11 EBV negative and one (case 654) EBV positive] and four non-mediastinal cases are included. The 20 most common genes with alterations are illustrated. Abbreviations: CHL, classic Hodgkin lymphoma; LBCL, large B-cell lymphoma; No., number; TMB, tumor mutational burden

Twenty-nine cases were submitted to the workshop with a diagnosis of GZL. The following had histologic and immunophenotypic features consistent with GZL: 19 EBV-negative cases (with mediastinal involvement: cases 165, 317, 327, 368, 372, 436, 460, 479, 481, 530, 565, 701, 751, and 761, and the initial diagnostic biopsy of case 189 also included with composite/sequential cases discussed below; without mediastinal involvement: cases 332, 352, and 519; case 774 was included with non-mediastinal cases given the presence of intrathoracic lymphadenopathy without a clearly defined thymic/anterior mediastinal mass), seven EBV-positive cases (with mediastinal involvement: cases 654 and 577; without mediastinal involvement: cases 135, 210, 320, 555, and 610), and one FL-related case (750, non-mediastinal). Clinical details and available molecular investigations are reported for 19 of the GZL cases in Table 1 (includes all 18 EBV-negative cases and case 189). Lymph3Cx GE analysis was performed on 17 cases that had available material (Fig. 4). Sixteen cases (12 EBV-negative MGZLs, one EBV-positive MGZL, and four EBV-negative non-mediastinal cases) were analyzed with a capture-based targeted next generation sequencing panel of 146 genes recurrently mutated in B cell lymphomas (Fig. 5; Supplementary Methods; Supplementary Table 1). Six cases also had mutation data provided by the case submitters (Supplementary Table 2).

**Table 1:** Clinicopathologic features of 19 EBV-negative GZLs

	MGZL	NMGZL	Total
Number	15^a^	4^b^	19
Age in years (range)	40 (13–77)	58 (48–66)	49 (13–77)
Male/female	9/6	3/1	12/7
Clinical stage			
I–II	6/9	0/3	6/12
III–IV	3/9	3/3	6/12
Ig monoclonality	2/3	3/3	5/6
*BCL2*-R^c^	0/14	1/4	1/18
*BCL6*-R^c^	0/15	1/3	1/18
*MYC*-R	0/4	0/3	0/7
*PDL1/2* gain/amp^d^	2/2	1/1	3/3
*REL* gain/amp	nd	0/1	0/1
*CIITA*-R	0/1	0/1	0/2
Lymph3Cx			
PMBL-sig	9	1	10
DLBCL-sig	0	2	2
Unclear-sig	3	1	4

*Abbreviations*: *EBV* Epstein-Barr virus, *GZL* gray zone lymphoma, *MGZL* mediastinal GZL, *NMGZL* non-mediastinal GZL, *Ig* immunoglobulin, *R* rearrangement, *amp* amplification, *PMBL* primary mediastinal large B-cell lymphoma, *DLBCL* diffuse large B cell lymphoma, *sig* signature, *nd* not determined

^a^9/15 MGZL cases also had extramediastinal disease

^b^Sites of disease included abdominal, axillary, and thoracic lymph nodes, and bone

^c^*BCL2* rearrangement detected in case 352 and *BCL6* rearrangement identified in case 774

^d^*PDL1/2* gains/amplification detected in cases 189, 372, and 519

### Cases illustrating challenges to defining the diagnostic border between CHL and MGZL

**Fig. 6:**
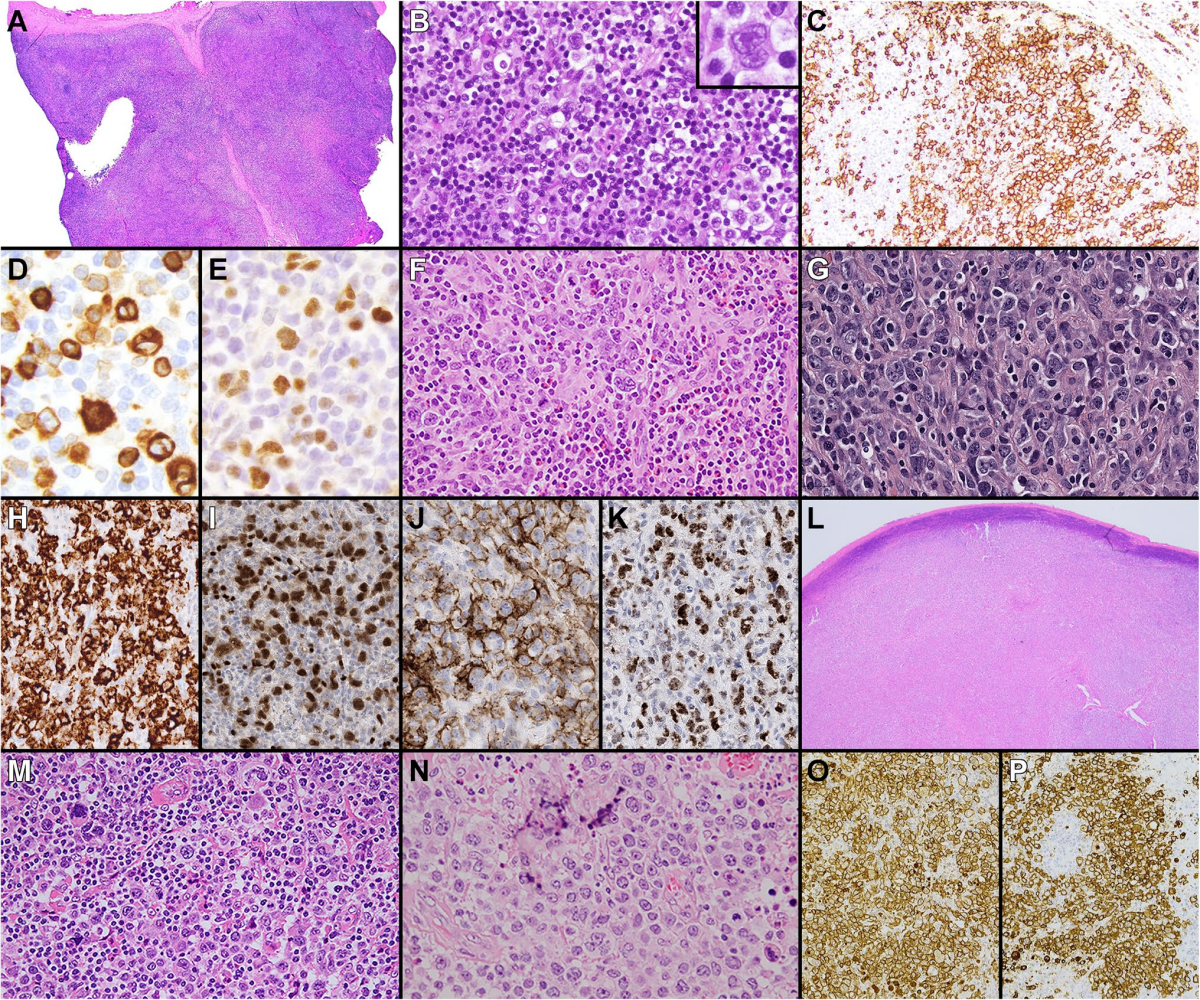
EBV-negative MGZLs with CHL-like features (cases 701, 317, and 751). Case 701 showed a vaguely nodular proliferation with focal fibrotic bands (**A**) reminiscent of CHL (**B**). The neoplastic cells demonstrated strong expression of CD20 (**C**) and CD79a (**D**), with moderate expression of OCT2 (**E**). Rearrangements of *BCL2* and *BCL6* genes were not detected by FISH in this case and Lymph3Cx analysis provided an unclear GE. Case 317 demonstrated a vaguely nodular proliferation of pleomorphic large cells, with some areas more closely resembling CHL (**F**), LBCL (**G**), or with transitional features. The neoplastic cells showed strong CD30 (**H**) and PAX5 (**I**) expression, and positivity for CD20 (**J**) and OCT2 (**K**). Lymph3Cx analysis showed a PMBL GE signature. Case 751 showed a diffuse growth (**L**), with some areas more closely resembling CHL (**M**), and other areas resembling LBCL, including clearly centroblast-like cells (**N**). The lymphoma demonstrated strong positivity for CD30 (**O**) and CD15 (**P**). Lymph3Cx analysis provided a PMBL GE signature

Ten cases submitted to the workshop (cases 165, 189, 317, 327, 372, 460, 479, 481, 701, and 751), all of which had mediastinal involvement and were EBV negative, corresponded to LYSA groups 0–1 [1] or to CHL-like/intermediate subtypes of MGZL described by other authors (Fig. 4A; Supplementary Fig. 2) [15, 30–32]. Case 701 (N. Barasch, University of Pittsburgh Medical Center, USA) represented the only MGZL case with LYSA group 0/CHL-like features and showed diffuse, moderate to strong expression of all B cell markers (Fig. 4A; Fig. 6A–E). At least a subset of the large cells also appeared weakly BCL6 positive, and some were suspicious for CD45 positivity. It is worth mentioning that despite a vague nodularity and some extent of fibrosis, the typical NSCHL architecture was not observed in this case although the tumor density was low. In the centrally reviewed case series reported by Pilichowska et al. [3], the typical architectural features of NSCHL and low tumor density would favor a diagnosis of CHL (with or without a full B cell immunophenotype) rather than GZL. In spite of the low number of tumor cells present in case 701, the panel agreed with the submitted diagnosis of MGZL for the diffuse and mostly strong expression of all B cell markers in the neoplastic cells, corresponding to LYSA group 0 per Sarkozy et al. [1]. Although the number of LYSA group 0 cases evaluated in this previous study was limited, GE analysis did show that such cases separately cluster from CHL [34]. CHL-like cases with strong expression of many/all B cell markers and EBV negativity are a particular diagnostic challenge with significant therapeutic impact, and consensus diagnostic criteria would be desirable.

The nine remaining cases confirmed as MGZL showed CHL-like/intermediate or LYSA group 1 morphology and included the initial diagnostic sample of sequential case 189 (A. Gupta, Memorial Sloan Kettering Cancer Center, USA), which showed composite areas of CHL and LBCL, as well as additional transitional areas. Case 317 (S. Lazzi, University of Siena, Italy) similarly showed areas more closely resembling CHL or LBCL, or with transitional features, strong CD30 expression, partial CD45, and preservation of the B cell program (Fig. 4A; Fig. 6F–K). Of particular challenge were cases showing very wide sheets of neoplastic cells for which the differentiation from the NS2 type and/or syncytial variants of CHL is mandatory due to the important therapeutic implications (cases 165 [H. Shao, H. Lee Moffitt Cancer Center, USA] and 751 [J. Hon, LAC + USC Medical Center, USA]). NS2 type CHL is defined by the presence of at least one of three criteria, the third of which indicating that more than 25% of the nodules contain numerous bizarre and highly anaplastic-appearing HRS cells without depletion of lymphocytes [39], thus bordering the GZL concept. The syncytial variant of CHL has been less well-defined, but basically consists of CHL cases with a high number of tumor cells growing cohesively with a confluent distribution [40–43]. The typical NSCHL growth pattern may be missing or only minimally represented in syncytial variant CHL, which further increases the diagnostic difficulty. This discrimination can be even more complex in patients with relapsing/refractory CHL, which is often characterized by very abundant neoplastic cells that may additionally acquire CD20 expression. A suggested guiding parameter is cell cytology, which tends to be enriched with centroblast-like mononuclear cells in GZL compared to NS2/syncytial CHL, which has a predominant HRS cell type [1, 3, 43]. A high degree of cellular pleomorphism and less conspicuous inflammatory background would also favor GZL rather than NS2/syncytial CHL [3, 30].

The expression of ≥ 3 B cell markers seems the standard in this group of cases, with a higher number of positive B cell markers required to diagnose MGZLs with CHL-like/intermediate morphologic features compared to cases with LBCL morphology (Fig. 4A). Eight of nine cases showed expression of five to six B cell markers, with wide variability in the percentage of positive cells and staining intensity (Supplementary Fig. 2). While CD20 and PAX5 mostly showed moderate to strong staining, CD79a, OCT2, BOB1, and CD19 varied from strong to weaker expression. CD30 was positive in all cases, usually in > 75% of neoplastic cells (70% of cases), CD15 was positive in six of nine cases (four cases with an average of 50% positive neoplastic cells), CD23 was partially positive in four of eight cases, and BCL6 was expressed by three of eight cases (one case strongly positive). CD45 was not uniformly positive in this group of MGZL, with expression seen in only five of nine cases. NGS analysis of nine cases from this group showed mutations in genes commonly associated with CHL, PMBL, and MGZL (Fig. 5; Supplementary Table 1; Supplementary Table 2). Interestingly, four cases also showed mutations in genes related to the chromatin structure, such as members of the histone H1 family (*HIST1H1E, HIST1H1C*) and *KMT2C*; alterations in these genes were more frequent in MGZL cases with CHL-like features compared to those with LBCL-like histology.

Case 751 (J. Hon, LAC + USC Medical Center, USA) was debated amongst the EAHP-WS panel due to its immunophenotypic aberrancies compared to the other cases in this group. This case presented in a 29-year-old male with a history of latent tuberculosis, systemic symptoms, and a bulky multilobated anterior mediastinal mass with associated cervical and bilateral supraclavicular lymphadenopathy. The morphologic features of the case fit well within LYSA group 1 (CHL-like with transitional and PMBL-like areas), but all neoplastic cells displayed only weak to moderate PAX5 expression among the B cell markers (negative for CD20, CD79a, OCT2, BOB1, and CD19), and uniform CD30 and CD15 positivity (Fig. 4A; Fig. 6L–P). A syncytial CHL was considered, but finally a diagnosis of MGZL was favored due to the presence of sheets of frankly centroblastic-appearing cells in some areas. Conversely, a diagnosis of refractory/relapsing syncytial CHL was favored over GZL for cases 340 (Y. Al-Ghamdi, Weill Cornell Medicine, USA) and 792 (C. Lome-Maldonado, Instituto Nacional de Cancerologia, Mexico). Both occurred in patients previously diagnosed with CHL (case 340 also showed a clonally related FL); showed extensive syncytial areas with predominantly HRS-like cells that were positive for CD30, CD15, and PAX5; were negative or only minimal positive for CD20; and showed expression of two or one additional B cell marker, respectively.

### Cases illustrating the diagnostic border between PMBL and MGZL

Five cases submitted to the workshop (cases 368, 436, 530, 565, and 761), all of which had mediastinal involvement and were EBV negative, corresponded to LYSA groups 2–3 [1] or to the LBCL-like subtype of other reports [30–33]. Cases 565 (I. Shupletsova, National Research Center for Hematology, Russian Federation) and 761 (M. Pugh, University of Birmingham, UK) could be morphologically classified as LYSA group 3, as MGZLs at the extreme border with PMBL. B cell marker expression was frequently lost or only present in subsets of neoplastic cells, often at a weaker intensity than expected for a LBCL (Fig. 4A; Supplementary Fig. 2). CD20 was positive in only one of five cases, OCT2 was partially positive in two of four cases, and CD79a was partially positive in two of five cases. All cases evaluated were negative for BOB1 and CD19. PAX5 was the exception to this and was positive in four of five cases, with strong to moderate staining intensity in most. CD30 was diffusely positive (> 75% cells) in all cases, and CD15 was positive in three of five. CD45 was positive in only one of five cases, an observation that suggests that the role of this antigen in the diagnosis of GZL, as is recommended by the WHO-HAEM4R classification [2], should be reconsidered.

Given that some degree of immunophenotypic aberrancy is tolerated in PMBL/DLBCL, the question arises as to what extent of aberrancy is required to distinguish GZL (LBCL-like) from PMBL/DLBCL. This especially pertains to those GZLs fulfilling the criteria for LYSA group 3 according to Sarkozy et al. [1]. Although a full panel of B cell markers was not available in all PMBL cases submitted to this session, most showed expression of ≥ 3 B cell markers, including CD20 in the vast majority (Fig. 1). Some degree of CD15 expression (weak or golgi/dot-like) was seen in only two cases (169 [K. Karube, University of the Ryukyus, Japan] and 264 [S. Weindorf, Stanford University, USA]), while the remaining cases were negative. Conversely, three of five (60%) MGZL of this group were CD15 positive (> 75% neoplastic cells in two cases), and none showed only golgi/dot-like staining. In this light, cases with a LBCL-like morphology that are negative for CD20 and positive for CD15, regardless of the type of positivity, must be investigated for all B cell associated markers and CD30 before ruling out a PMBL/DLBCL. In such cases, negative staining for other B cell markers and diffuse, preferably strong, CD30 positivity with CD15 expression would favor a diagnosis of MGZL.

### Cases submitted to the workshop as possible non-mediastinal/non-thymic GZL

The MGZLs submitted to the EAHP-WS mostly developed in younger patients (average age 40 years; range 13–77 years) and at lower stages (stage I/II in six of nine cases and no stage IV disease) compared to the non-mediastinal GZLs (cases 332, 352, 519, and 774), which presented in older individuals (average age 58 years; range 48–66 years) and advanced stages (stage IV in all three with available data) (Table 1), which is concordant with the prior literature [1–3, 30, 32, 35]. With recently published GE and sequencing analyses of GZLs, we are now increasingly aware that the site of disease onset makes a difference in terms of the underlying genetics of these lymphomas [34, 35]. There seems to be a clear thymic niche B-cell lymphoma spectrum encompassing both PMBL and MGZL, from which the non-thymic cases should be separated despite overlapping morphology and immunophenotype [34, 35]. Specifically, by applying Lymph3Cx analysis, which is trained to differentiate PMBL from DLBCL, MGZLs and PMBLs demonstrate similar GE signatures [8, 34, 35]. Lymph3Cx analysis was performed on 16 GZLs submitted to the EAHP-WS (Table 1; Fig. 4). Nine of 12 (75%) EBV-negative MGZLs showed a PMBL-like GE signature (six had CHL-like/intermediate and three LBCL-like morphology) of which eight also stained diffusely positive for PDL1. The remaining three MGZL (cases 327 [A. Owczarczyk, University of Michigan, USA], 479 [M. Takeda, University of Southern California, USA], and 701 [N. Barasch, University of Pittsburgh Medical Center, USA]; all with CHL-like/LYSA groups 0–1 morphology) showed an “unclear” GE signature with values borderline between PMBL and DLBCL.

**Fig. 7:**
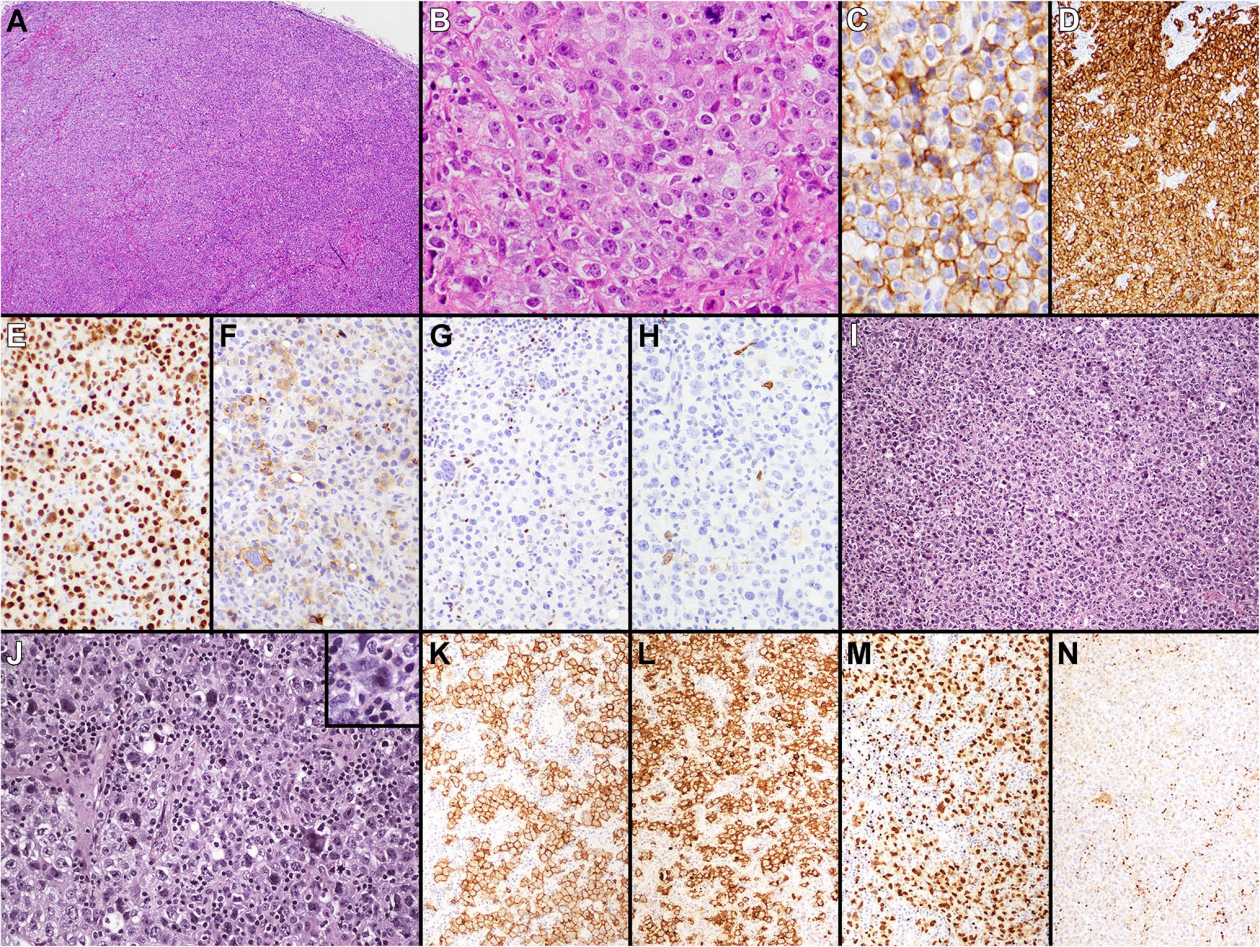
EBV-negative GZLs with LBCL-like features (cases 332 and 774). Case 332 showed a vaguely nodular (**A**) proliferation of predominantly large mononuclear cells that had pale/clear cytoplasm (**B**). The neoplastic cells were diffusely positive for CD45 (**C**), CD30 (**D**), and PAX5 (**E**), while few were positive for CD15 (**F**), and they were negative for OCT2 (**G**) and CD20 (**H**). Lymph3Cx analysis showed a DLBCL GE signature and sequencing demonstrated mutations in *BCL2* and *TP53*. FISH studies showed no rearrangements of *MYC, BCL2*, or *BCL6.* Case 774 demonstrated a diffuse infiltrate of predominantly large mononuclear cells (**I**), with some showing HRS features (**J**). The neoplastic cells were strongly positive for CD30 (**K**) and CD15 (**L**), PAX5, at moderate to strong intensity (**M**), and were negative for OCT2 (**N**). RT-MLPA analysis was consistent with a PMBL GE signature, while Lymph3Cx analysis showed an unclear result

Of the four non-mediastinal GZLs, cases 332 (S. Sadigh, Massachusetts General Hospital, USA) (Fig. 7A–H) and 352 (C. Bertuzzi, IRCCS AOUBO, Italy) had a DLBCL-like GE signature (both had LBCL-like morphology) (Fig. 4B). NGS identified mutations of *BCL2* and *TP53* in case 332 (Fig. 5; Supplementary Table 2), a mutational profile more commonly seen in the GCB subtype of DLBCL compared to PMBL, CHL, or MGZL [35]. Case 352 also showed a *BCL2* rearrangement with FISH analysis and a mutation in *KMT2C* (Table 1; Fig. 5), further supporting a more appropriate diagnosis of DLBCL (possibly transformed from an undetected FL) with a defective B cell phenotype. Case 519 (R. Ryan, University of Michigan, USA) conversely showed a PMBL-like GE signature (Fig. 4B). This lymphoma presented in a 58-year-old immunocompetent male with lung involvement and extramediastinal lymph node enlargement, and showed CHL-like/intermediate morphology with a variable B cell immunophenotype that would suggest possible non-mediastinal GZL. However, a T-cell lymphoproliferative disorder was detected in the patient’s peripheral blood (no additional data available), which makes it difficult to consider this lymphoma as a prototypical GZL. Case 774 (M. Parrens, CHU Bordeaux, France) (Fig. 7I–N) arose in a 66-year-old-female with multiple spinal osteolytic lesions and intrathoracic lymphadenopathy, but did not show a definite anterior mediastinal/thymic mass, and was therefore considered as non-mediastinal. The biopsy showed a diffuse infiltrate of predominantly large mononuclear cells, with some showing HRS features. The lymphoma had a striking LBCL-like morphology; the neoplastic cells were strongly positive for CD30 and CD15, showed markedly diminished expression of the B cell program (negativity for CD20, CD79a, OCT2, BOB1, and CD19) (Fig. 4B), as well as for CD45, CD23, and PDL1. Only PAX5 was positive at moderate to strong intensity, and there was partial, weak to moderate expression of MAL. This case had discordant results with GE profiling studies. While the RT-MLPA assay [4], which uses three genes to identify PMBL (*FCER2*/CD23, *TNFRSF8*/CD30, and *MAL*), showed a PMBL GE signature, the Lymph3Cx assay [8], which uses 30 genes to discriminate PMBL from DLBCL, showed an “unclear” GE signature. Given the larger number of genes analyzed by Lymph3Cx, the “unclear” result is arguably more reliable than the PMBL signature attributed by RT-MLPA, which is likely impacted by the strong expression of CD30 and MAL proteins by this lymphoma. A *BCL6* gene rearrangement and gains of *CIITA* were detected by FISH, while sequencing studies showed both *CIITA* and *NOTCH2* mutations in this case (Fig. 5; Supplementary Table 1; Supplementary Table 2). The three non-mediastinal GZLs tested for PDL1 were negative.

In summary, the MGZLs in this session presented in younger patients at lower clinical stages, frequently showed PDL1 positivity, and demonstrated a PMBL-like GE signature in 75% of cases, in contrast with the non-mediastinal GZLs, which developed in older patients with more advanced clinical stages, and showed a DLBCL-like GE signature in at least 50% of samples. Mutations in genes commonly reported in PMBL and CHL were detected in most mediastinal cases and appeared less frequent in cases without mediastinal involvement, although the latter group was limited to only four cases. Recognizing the limitations of this small case series, the data from this session of the EAHP-WS supports the concept that LBCLs originating within the thymic niche represent a biologic spectrum of diseases distinct from those lymphomas arising outside of this anatomic location, even though they may exhibit similar morphologic and immunophenotypic features. As mentioned previously, this issue has been clarified in the recent ICC and WHO-HAEM5 classifications [16, 17]. Accordingly, at least cases 332, 352, and 519 among the non-mediastinal GZLs should be classified as DLBCL and NOS (with GZL-like features). The re-classification of case 774 as a DLBCL still remains debatable since no B cell markers were detected except PAX5.

## EBV-positive mediastinal B cell lymphomas

Session 2 of the EAHP-WS, dedicated to CHL and its mimics, also examined the issue of EBV-positive B cell lymphoproliferative disorders, and most EBV-positive cases submitted to the workshop were addressed during this session. Among the cases submitted as PMBL and GZL, EBV was positive in 7 (PMBL: case 452; MGZL: cases 577 and 654; non-mediastinal GZL: cases 135, 210, 320, and 610), all of which arose in immunocompetent individuals. Case 555 (L. Massoth, Massachusetts General Hospital, USA), which had features of a GZL, developed in the immunodeficiency setting and is excluded from this discussion.

EBV infection is known to modulate the immunophenotype and mRNA expression profiles of infected neoplastic cells, which contributed to the recognition of a specific category of EBV-positive DLBCL, NOS in the WHO-HAEM4R classification [2]. These EBV-positive DLBCLs include a heterogenous set of histologic patterns, from a diffuse proliferation of large B cells to a T cell/histiocyte-rich/polymorphic pattern with scattered large B cells. This latter group of EBV-positive DLBCLs likely also includes at least a subset of reported EBV-positive B cell lymphomas with features of GZL [44, 45]. EBV-positive PMBLs and GZLs are quite rare, but remained recognized in the WHO-HAEM4R classification [2]. However, the question remains if such cases, and in particular those arising outside of the mediastinum, are better included in the category of EBV-positive DLBCL, NOS at this time [1–3, 35, 44, 45]. Given the rarity of such cases, the availability of GE and genetic data specifically focusing on EBV-positive PMBL and GZL is very limited [34, 35]. In recent publications, the EBV-positive cases with GZL features for the most part showed a distinct GE signature, supporting their segregation from EBV-negative GZL [34, 35]. The four non-mediastinal EBV-positive cases with GZL-like features included in the session (cases 135, 210, 320, and 610) were classified as EBV-positive DLBCL, NOS by the EAHP-WS panel, given the considerations detailed above. Lymph3Cx GE data was available for two of these cases and none showed a PMBL signature (case 610 [A. Nicolae, Hautepierre Hospital, France] showed a DLBCL GE signature and case 210 [S. Dirnhofer, University Hospital Basel, Switzerland] showed an unclear GE signature).

**Fig. 8:**
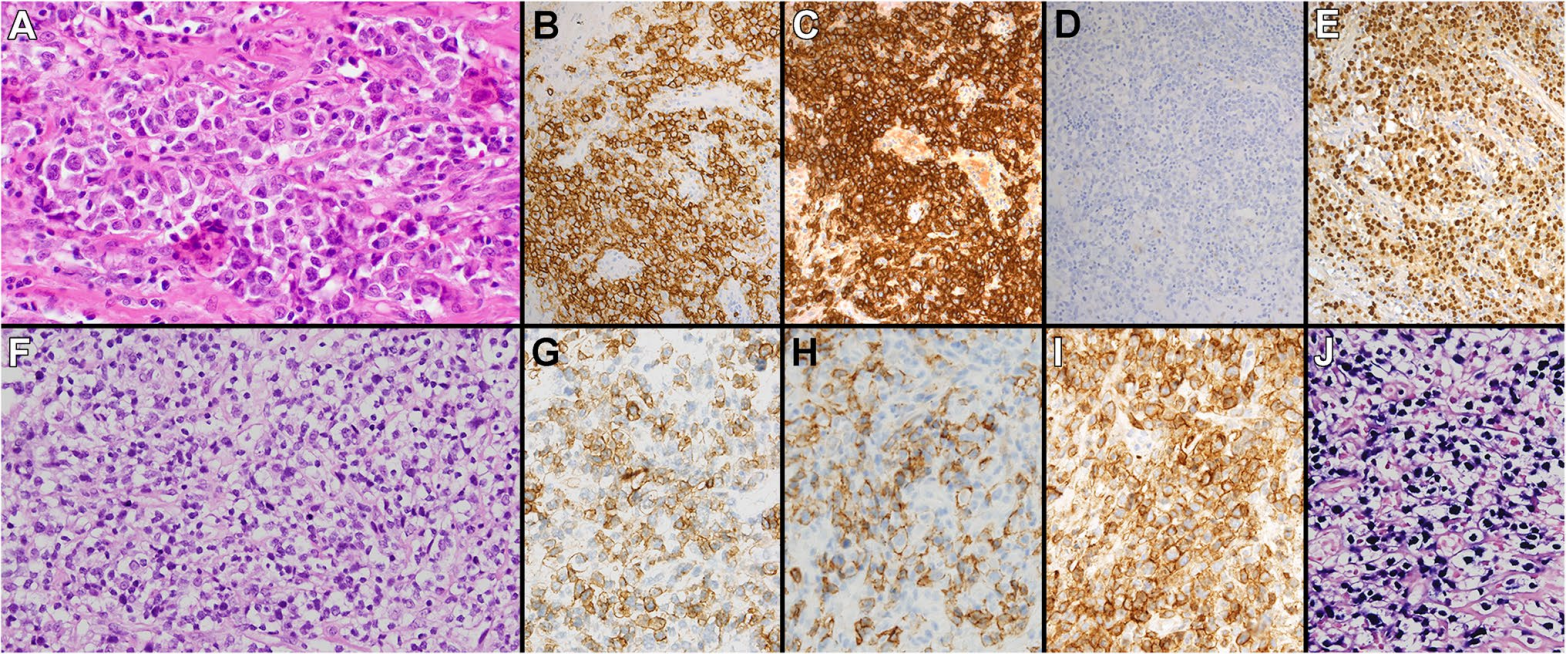
EBV-positive MGZL (case 654) and PMBL (case 452). Case 654 showed GZL-like morphology (**A**), strong positivity for CD20 (**B**) and CD30 (**C**), negativity for CD15 (**D**), and diffuse EBER positivity (**E**). Case 452 demonstrated a morphology (**F**) and immunophenotype compatible with PMBL, showing expression of CD30 (**G**), CD23 (**H**), and PDL1 (**I**), in addition to diffuse EBER (**J**) staining

However, rare EBV-positive cases consistent with MGZL were found to share some GE and genetic overlap with EBV-negative PMBL and MGZL [34, 35]. Although this data is limited and preliminary, it supports the possible inclusion of EBV-positive PMBL or MGZL within the spectrum of mediastinal/thymic B cell lymphomas. Case 654 (A. Bogusz, University of Pennsylvania, USA) was very representative of this issue and fit well with the concept of a mediastinal/thymic B cell lymphoma profile in spite of EBV integration. This lymphoma arose in a 22-year-old immunocompetent male with an isolated mediastinal mass, showed MGZL-like morphology and immunophenotype (strong CD30 and CD20 expression; negative for CD15, CD79a, OCT2, and BOB1), and demonstrated a PMBL-like GE with Lymph3Cx analysis (Fig. 4A; Fig. 8A–E). As further support to the hypothesis of an EBV-positive MGZL, NGS analysis revealed mutations in *B2M, CIITA, GNA13, SOCS1, TNFAIP3*, and *XPO1* genes (Fig. 5; Supplementary Table 1). Case 577 (D. Fong, Kaiser Permanente San Rafael, USA), which showed morphologic and immunophenotypic features at the border between PMBL and MGZL, had no GE or NGS data available. Case 452 (A. Nicolae, Hautepierre Hospital, France) (Fig. 8F–J), arising in a 31-year-old immunocompetent female with an anterior mediastinal mass, was morphologically and immunophenotypically compatible with PMBL (including MAL staining), and showed diffuse positivity for EBER, LMP1 and EBNA2. RT-MLPA analysis performed on this case showed an “unclassifiable” GE signature due to the presence of EBV. Additional specimen was not available from this case to perform further Lymph3Cx or NGS testing.

As a general rule, it still seems wiser to favor the diagnosis of EBV-positive DLBCL, NOS in EBV-positive cases that have a LBCL- or GZL-like morphology arising in non-mediastinal sites. One consideration would be to indicate the morphologic subtype of these EBV-positive DLBCL cases (e.g., PMBL-like, GZL-like), although there is no documented diagnostic or clinical relevance at this time. Regarding the interpretation of EBV positivity in MGZL cases, there is some limited evidence supporting their existence [34, 35]. This is also conceptually conceivable when considering the more established presence of EBV in other mediastinal B cell lymphomas, such as NSCHL or, more rarely, PMBL.

## PMBL-like LBCLs and GZLs associated with FL

It is well recognized that FL may transform into DLBCL, CHL, high-grade B cell lymphoma, B-lymphoblastic leukemia/lymphoma, and even histiocytic/dendritic cell neoplasms [2, 46, 47]. Case 193 (L. Rimsza, Mayo Clinic Scottsdale, USA), as detailed above in the PMBL section, and cases 490 and 750 may add PMBL-like and GZL-like transformations to this list. Case 490 (L. Wake, John Hopkins University, USA) was shown to have clonally related FL and GZL components, and the GZL in this case also harbored a *BCL2* rearrangement detected by FISH. Case 750 (J. Hon, LAC + USC Medical Center, USA) illustrated a non-mediastinal GZL associated with foci of in situ follicular neoplasia, with an IGH::*BCL2* fusion identified by FISH in the GZL component.

## Cases of composite or sequential CHL and PMBL/DLBCL and/or GZL submitted to the workshop

Seven cases were submitted to the EAHP-WS as composite (cases 196, 652, 760, and 811) or sequential (cases 189, 232, and 547) CHL and PMBL/DLBCL and/or GZL. In the diagnostic setting, the co-occurrence of a PMBL/DLBCL and a CHL, each with the appropriate morphology and immunophenotype, either in the same tissue or separate tissues, defines a composite lymphoma, regardless of which lymphoma comes first [2, 30]. No significant transitional areas should be observed in such cases. Although the composite or sequential lymphomas may be clonally related, this finding does not support the classification of such cases as GZL in the WHO-HAEM4R [2].

The group of composite/sequential cases submitted to the EAHP-WS raised some issues for debate. During EAHP-WS panel discussions, it was noted that proper classification may be challenging in composite cases, particularly if the lymphomas are synchronous, when the CHL and LBCL areas show immunophenotypic overlap (case 460 [P. Adhikari, University of Vermont Medical Center, USA]) or when morphologic transitional/intermediate areas are detected in serial tissue sections (cases 189 [A. Gupta, Memorial Sloan Kettering Cancer Center, USA] and 460). Such features would understandably lead one to consider the diagnosis of GZL, and would explain the not infrequent use of “composite GZL” terminology, which is not provided in the WHO-HAEM4R classification or proposed WHO-HAEM5 and ICC classifications [2, 16, 17]. Also noteworthy is the observation that in both composite and sequential cases, one of the two lymphomas may be represented by a GZL (cases 189 and 547 [P. Basra, H. Lee Moffitt Cancer Center, USA]). Case 189 arose as a GZL (case also included with the GZL group) and subsequently relapsed as CHL.

## Summary

In session 3 of the 20th meeting of the European Association for Haematopathology lymphoma workshop we observed cases that spanned the spectrum between PMBL, MGZL, and CHL. The EAHP-WS panel diagnoses for all cases included in this session may be viewed in Supplementary Table 3. Our session emphasized that PMBLs may demonstrate morphologic and immunophenotypic heterogeneity, which requires evaluation of additional pan-B- cell or PMBL-associated markers. In particular, unusual features such as extensive necrosis, a more polymorphous appearance, expression of immunoglobulin light chains, or plasmacytoid differentiation may be occasionally observed in otherwise classic PMBL. Rare cases may exhibit loss of CD20 expression, and evaluation of other pan-B cell antigens is required to exclude other lymphomas including CHL and MGZL. Likewise, CD15 positivity may uncommonly be observed in PMBL, more often with partial and/or a paranuclear dot-like appearance. If neoplastic cells are diffusely and strongly positive for CD15, a MGZL must instead be considered. Some PMBLs may also show only minimal CD30 and/or CD23 staining, and evaluation of other PMBL-associated markers, including MAL, PDL1, PDL2, or CD200, is useful to support the diagnosis in the appropriate clinical setting. Additional genetic and molecular profiling assays are increasingly available and may help support the diagnosis of PMBL over a mediastinal DLBCL in equivocal cases. PMBLs recurrently show gains of chromosome 2p16.1/*REL*; gains/amplification of 9p24.1/*JAK2*; inactivating mutations of *TNFAIP3, NFKBIE, SOCS1,* and *PTPN1*; gain of function mutations in *STAT6*; rearrangements and/or mutations of *CIITA*; alterations of *B2M* and *CD58* [2, 20, 21, 25]; and more frequently a PMBL GE signature with Lymph3Cx analysis [8]*.*

The diagnostic borders between MGZL and CHL or PMBL remain difficult to define and, in light of the biological spectrum, are largely arbitrary. A diffuse, sheet-like growth pattern of neoplastic cells should not automatically place a case that otherwise has cytologic and immunophenotypic features of CHL into the MGZL category. Identification of cells with more centroblastic cytology and loss of typical CHL architecture and inflammatory background may favor a diagnosis of MGZL. It is important to evaluate multiple B- cell markers (6 were applied to most cases of GZL if material was available) and to not only rely on CD20 expression when differentiating MGZL from CHL or PMBL/DLBCL. Defined cut-offs for percentage of positive cells or staining intensity cannot be assessed, although most CHL-like MGZL in the studied series showed positivity in more than 50% neoplastic cells at moderate to strong intensity.

Special mention is required for cases that would fit within LYSA group 0 of Sarkozy et al. [1], which includes cases histologically resembling typical CHL that express uniform and strong CD20 and other B cell markers (such as OCT2, BOB1, PAX5, and/or CD79a). Limited data is available and the inclusion of these rare cases in the category of MGZL rather than CHL is still controversial. Eleven such cases that were previously studied were shown to have a GE profile that clustered separately from that of CHL, although the separation was quite small [34]. At present both the ICC and WHO-HAEM5 classifications recommend a high tumor cell density for the diagnosis of MGZL [16, 17]; therefore, LYSA group 0 type cases would most likely include lymphomas with features of NS2 type/syncytial CHL that show expression of a full or almost full B cell program at moderate to strong intensity. Nonetheless, WHO-HAEM5 does still recognize less common cases with only scattered HRS-like cells and an absence of confluent neoplastic cells [16], as are some of the cases included in the study published by Sarkozy et al. [1] and case 701 presented at the EAHP-WS. Further studies are needed to clarify the best place for these rare lymphomas within the spectrum of mediastinal B cell lymphomas. In general, since MGZLs show a heterogenous combination of morphology and immunophenotype, the diagnosis should not rely on a single feature or marker, and equivocal cases should always be discussed in multidisciplinary meetings in the light of potential therapeutic options.

Although the diagnosis of PMBL-like lymphomas outside of the mediastinum remains debatable, there is increasing evidence that extramediastinal GZL-like lymphomas are not biologically related to MGZL and should instead be considered within the category of DLBCL, NOS [34, 35]. Rare PMBLs and MGZLs associated with EBV may exist, but EBV-positive GZL-like lymphomas outside of the mediastinum should be considered EBV-positive DLBCL, NOS at this time [16, 17]. Composite or sequential lymphomas with CHL and PMBL/DLBCL may be challenging to evaluate, particularly in those cases that show areas of intermediate/transitional morphology or demonstrate an overlapping immunophenotype between the CHL and PMBL/DLBCL components that might lead one to favor a diagnosis of GZL.

### Supplementary Information

Below is the link to the electronic supplementary material.

Supplementary file1 (DOCX 25 KB)

Supplementary file2 (PDF 155 KB)

Supplementary file3 (PDF 303 KB)

Supplementary file4 (XLSX 59 KB)

Supplementary file5 (DOCX 18 KB)

Supplementary file6 (XLSX 13 KB)

## Data Availability

All original raw and processed gene expression and next-generation sequencing data will be made available for research purposes upon request by the first and senior authors.

## Declarations

LMR is a named inventor on a patent filed by the National Cancer Institute (“Methods for determining lymphoma type”).
