# Supplementary material for: Mediastinal large B cell lymphoma and surrounding gray areas: a report of the lymphoma workshop of the 20th meeting of the European Association for Haematopathology
Source: Virchows Arch. 2023 Aug 2;483(6):733–49. doi: 10.1007/s00428-023-03550-5 (PMC10700426; doi:10.1007/s00428-023-03550-5)
Supplement: Supplementary file 1 — Supplementary file1 (DOCX 25 KB) [file 428_2023_3550_MOESM1_ESM.docx]

**Mediastinal large B-cell lymphoma and surrounding gray areas: a report of the lymphoma workshop of the 20^th^ meeting of the European Association for Haematopathology**

Sarah E. Gibson^1^, Stefan Docjinov^2^, Snjezana Dotlic^3^, Sylvia Hartmann^4^, Eric D. Hsi^5^, Monika Klimkowska^6^, Socorro Maria Rodriguez-Pinilla^7^, Thomas A. Tousseyn^8^, Lisa M. Rimsza^9^, Colleen A. Ramsower^10^, Karen Rech^11^, Stefano A. Pileri^12^, Daphne de Jong^13^, Elena Sabattini^14^

1. Division of Hematopathology, Department of Laboratory Medicine and Pathology, Mayo Clinic, Phoenix, AZ, United States; ORCID ID: 0000-0003-2427-6442
2. Department of Pathology, Morriston Hospital, Swansea Bay University Health Board, Swansea, United Kingdom
3. Department of Pathology and Cytology, University Hospital Centre Zagreb, Zagreb, Croatia; ORCID ID: 0000-0002-2036-9206
4. Dr. Senckenberg Institute of Pathology, Goethe University Frankfurt am Main, Frankfurt am Main, Germany; ORCID ID: 0000-0003-3424-1091
5. Department of Pathology, Wake Forest University School of Medicine, Winston-Salem, NC, United States; ORCID ID: 0000 0001 8623 4067
6. Department of Clinical Pathology and Cancer Diagnostics, Karolinska University Hospital, Stockholm, Sweden; ORCID ID: 0000-0002-3391-930X
7. Pathology Department, Hospital Universitario Fundación Jiménez Díaz, Madrid, Spain; ORCID ID: 0000-0002-2191-1327
8. Department of Imaging and Pathology, Translational Cell and Tissue Research Lab, KU Leuven, Leuven, Belgium; ORCID ID: 0000-0002-0397-1086
9. Division of Hematopathology, Department of Laboratory Medicine and Pathology, Mayo Clinic, Phoenix, AZ, United States; ORCID ID: 0000-0003-1268-2750
10. Department of Research, Mayo Clinic, Scottsdale, AZ, United States; ORCID ID: 0000-0002-4991-7712
11. Department of Laboratory Medicine and Pathology, Mayo Clinic, Rochester, MN, United States; ORCID ID: 0000-0001-6770-2339
12. Division of Haematopathology, IEO, European Institute of Oncology IRCCS, Milan, Italy; ORCID ID: 0000-0001-8032-5128
13. Department of Pathology, Amsterdam UMC, location VUMC, Amsterdam, The Netherlands; ORCID ID: 0000-0002-9725-4060
14. Haematopathology Unit, IRCCS Azienda Ospedaliero-Universitaria di Bologna, Bologna, Italy; ORCID ID: 0000-0002-4136-3591

**Corresponding Author:**

Elena Sabattini, MD
Haematopathology Unit, IRCCS Azienda Ospedaliero-Universitaria di Bologna
Block 8 - Via Massarenti 9
40138 Bologna, Italy
[elena.sabattini@aosp.bo.it](mailto:elena.sabattini@aosp.bo.it)
0039-51-2144562

**Supplementary Methods**

***Targeted DNA Sequencing***

Tumor genomic DNA (gDNA) from 16 EBV-negative gray zone lymphomas was analyzed using a capture-based targeted sequencing panel, including coding exons and splice sites of 146 genes recurrently mutated in B-cell lymphomas (Table 1). DNA was quantified using a fluorometric method, with the Qubit dsDNA HS Assay Kit (Invitrogen) according to the manufacturer’s protocol.

*Criteria used to design the targeted gene panel*

The targeted sequencing panel was designed to include coding exons or hotspot regions of 146 genes that are recurrently mutated in B-cell lymphomas. The gene panel was optimized as follows:

1. Genes were chosen if recurrently mutated in >5% of B-cell lymphomas;
2. For genes with a well-defined hotspot, only the regions that included known mutations were covered;
3. For genes lacking a well-defined hotspot, all coding exons were covered.

The SeqCap EZ Choice Library (NimbleGen, Roche Diagnostics) probe system was designed to capture target regions.

*Library preparation and ultra-deep sequencing*

DNA libraries were prepared starting from at least 200ng of gDNA, which were sheared through sonication (Covaris) before library construction to obtain 200bp fragments. Libraries were then generated using the Kapa Library Preparation Kit (Kapa Biosystem) following the manufacturer’s instructions. Each library was labelled with a ‘barcode’, also called adapter, to allow recognition in the analysis phase. For a quality check, Bioanalyzer was used for fragment size control (200-500bp expected) and the Qubit dsDNA HS Assay Kit to quantify libraries. Enrichment of the regions of interest was obtained using a system of SeqCap EZ Choice Library probes (Roche Diagnostics Nimblegen). Final libraries were sequenced using the MiSeq (Illumina) instrument by paired-end sequencing (2x150 cycles). Multiplexed libraries were simultaneously sequenced following the manufacturer's procedure, along with an internal quality control (PhiX, Illumina). The number of libraries loaded into the sequencer was tailored to obtain at least >1000x coverage in 80% of the regions of interest.

*Bioinformatic pipeline*

Bioinformatic analysis was supported by SOPHiA Genetics (Service Analytical Platform for Clinical Genomics). A FASTQ file of each sample was uploaded to the SOPHiA DDM platform and the final output of SNVs and indels, generated according to a specific pipeline, were filtered applying the following criteria: I) at least 10 mutated reads (without strand bias); II) variant allele frequency (VAF) ≥ 0.05; III) frameshift and nonsense mutations or indels in coding regions and belonging to pathogenic or likely pathogenic classes according to Saphetor annotation; and IV) exclusion of variants with a population frequency ≥ 0.01 in the 1000 Genomes and ExAC databases.

**Table 1: Target gene panel**

| **Hotspots** | | | **All Coding Regions** | | | | | | | | | | | | | |
| --- | --- | --- | --- | --- | --- | --- | --- | --- | --- | --- | --- | --- | --- | --- | --- | --- |
| EZH2 | TBL1XR1 | TCF4 | ARID1A | BIRC3 | CD37 | CHD2 | EGR2 | HIST1H1E | IRF2BP2 | MAP3K14 | NXF1 | PTPN11 | SETD2 | TNFAIP3 | NFKBIL1 | PMAIP1 |
| MUC16 | TRRAP | ID2 | ASXL1 | BRAF | CD58 | CHEK2 | EP300 | HIST1H3B | IRF4 | MED12 | P2RY8 | RASFF1 | SIN3A | TNFRSF14 | RNU1-1 | SYK |
| NOTCH1 | WHSC1 | RHOA | ATM | BTG1 | CD70 | CIITA | FBXO11 | HVCN1 | IRF8 | MEF2B | PAX5 | RB1 | SOCS1 | TP53 | MCL1 | CELSR3 |
| NOTCH2 | XPO1 | DNMT3A | ATRX | BTK | CD79A | CREBBP | FBXW7 | ID3 | ITPKB | MGA | PCBP1 | RBM38 | SPEN | TRAF2 | SMARCA4 | DAZAP1 |
| NRAS | UBR5 |  | B2M | CARD11 | CD79B | CSF2RB | FGFR2 | IGLL5 | KLF2 | MS4A1 | PIK3CA | RIPK1 | SPI1 | TRAF3 | RELA | BAX |
| POT1 | MPEG1 |  | BCL2 | CCND1 | CDKN1A | CSMD3 | FOXO1 | IKBKB | KLHL6 | MYC | PIM1 | RPS15 | STAT3 | ZMYM3 | LTB | HNRNPH1 |
| PTPRD | EBF1 |  | BCL6 | CCND2 | CDKN1B | CXCR4 | GADD45B | IKZF3 | KMT2D | MYD88 | PLCG2 | RRAGC | STAT6 | ZNF217 | EWSR1 | PPM1D |
| RBMX | FAS |  | BCL7A | CCND3 | CDKN2A | DDX3X | GNA13 | IL4R | KRAS | NFKBIA | PRDM1 | S1PR2 | TCF3 | ZNF292 | KMT2C |  |
| SF3B1 | SWAP70 |  | BCOR | CD36 | CDKN2B | DTX1 | HIST1H1C | IRAK1 | MAP2K1 | NFKBIE | PTPN1 | SAMHD1 | TET2 | NFKBIZ | DUSP2 |  |
