# Supplementary material for: Mediastinal large B cell lymphoma and surrounding gray areas: a report of the lymphoma workshop of the 20th meeting of the European Association for Haematopathology
Source: Virchows Arch. 2023 Aug 2;483(6):733–49. doi: 10.1007/s00428-023-03550-5 (PMC10700426; doi:10.1007/s00428-023-03550-5)
Supplement: Supplementary file 3 — Supplementary file3 (PDF 303 KB) [file 428_2023_3550_MOESM3_ESM.pdf]

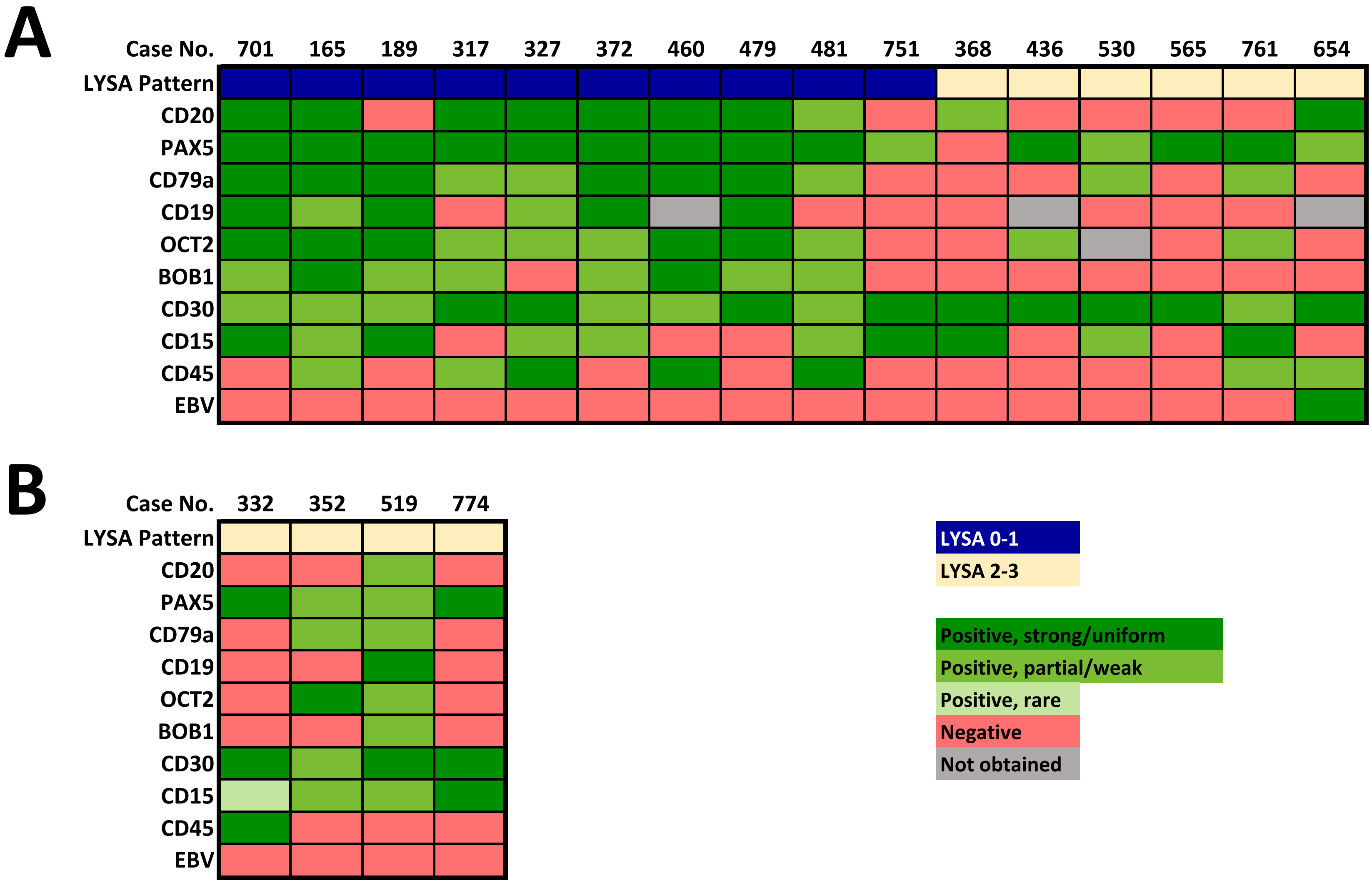

Supplementary Figure 2. Detailed immunophenotypes of 20 Workshop cases submitted as GZL. Detailed immunophenotypes, including intensity of staining, of sixteen MGZLs [15 EBV negative and one (case 654) EBV positive] (A) and four EBV-negative non-mediastinal cases (B) are illustrated. The morphologic classification of these cases into CHL-like/LYSA groups 0-1 or LBCL-like/LYSA groups 2-3 is included. Abbreviations: No., number.
