# Supplementary material for: Mediastinal large B cell lymphoma and surrounding gray areas: a report of the lymphoma workshop of the 20th meeting of the European Association for Haematopathology
Source: Virchows Arch. 2023 Aug 2;483(6):733–49. doi: 10.1007/s00428-023-03550-5 (PMC10700426; doi:10.1007/s00428-023-03550-5)
Supplement: Supplementary file 5 — Supplementary file5 (DOCX 18 KB) [file 428_2023_3550_MOESM5_ESM.docx]

| **Supplementary Table 2. Additional next generation sequencing data provided by case submitters^a^** | | | |
| --- | --- | --- | --- |
| **Case No.** | **Location** | **Histology** | **Genes Mutated** |
| 368 | Mediastinal | LBCL-like | *CCND2, CEBPA, TNFAIP3* |
| 372 | Mediastinal | CHL-like | *STAT6* |
| 479 | Mediastinal | CHL-like | *GNA13, TNFAIP3* |
| 332 | Non-mediastinal | LBCL-like | none detected (custom 111 gene panel) |
| 519 | Non-mediastinal | CHL-like | *ITPKB, NFKBIA, REL, RHOA, TET2* (MiOncoseq panel) |
| 774 | Non-mediastinal | LBCL-like | *CIITA, NOTCH2* |

Abbreviations: CHL, classic Hodgkin lymphoma; LBCL, large B-cell lymphoma; No., number

^a^Next generation sequencing platform is not specified for most cases (platform included in parentheses if available).
