# Supplementary material for: Mediastinal large B cell lymphoma and surrounding gray areas: a report of the lymphoma workshop of the 20th meeting of the European Association for Haematopathology
Source: Virchows Arch. 2023 Aug 2;483(6):733–49. doi: 10.1007/s00428-023-03550-5 (PMC10700426; doi:10.1007/s00428-023-03550-5)
Supplement: Supplementary file 2 — Supplementary file2 (PDF 155 KB) [file 428_2023_3550_MOESM2_ESM.pdf]

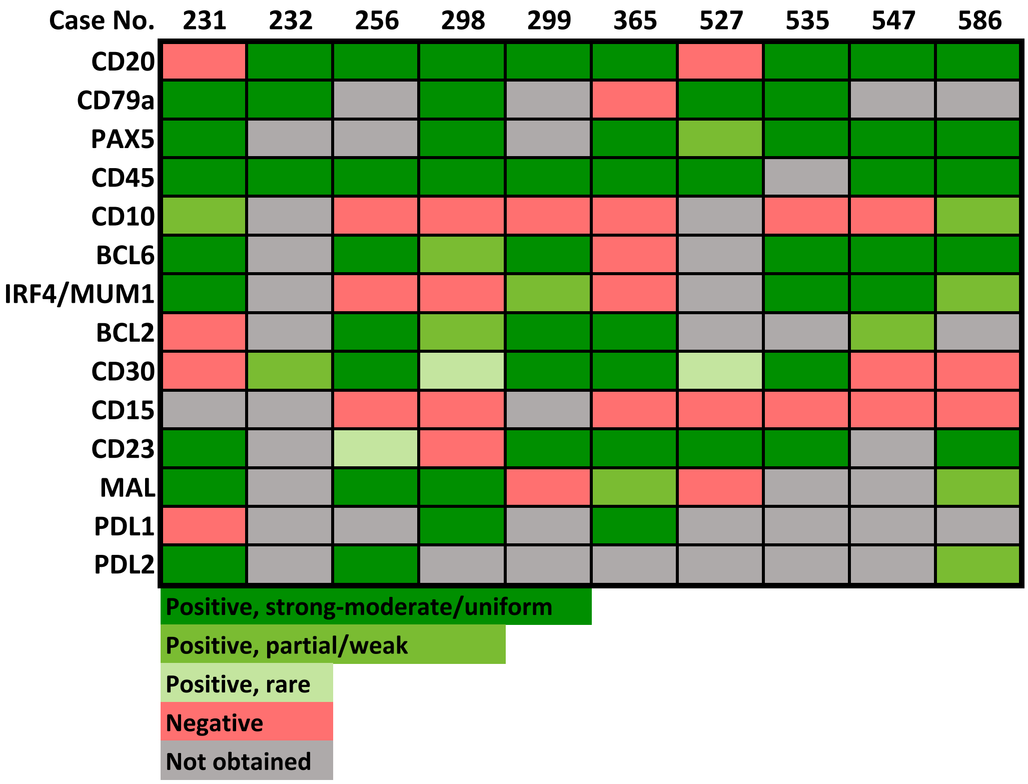

Supplementary Figure 1. Detailed immunophenotypes of 10 Workshop cases with features of classic PMBL. The figure illustrates detailed immunophenotypes, including intensity of staining, of each classic PMBL case. Abbreviations: No., number.
